# Gastric Schwannoma: A Rare but Important Differential Diagnosis of a Gastric Submucosal Mass

**DOI:** 10.1155/2012/280982

**Published:** 2012-08-08

**Authors:** William Yoon, Kari Paulson, Paul Mazzara, Sweety Nagori, Mohammed Barawi, Richard Berri

**Affiliations:** ^1^Department of Surgery, St. John Hospital and Medical Center, Detroit, MI, USA; ^2^Department of Pathology, St. John Hospital and Medical Center, Detroit, MI, USA; ^3^Department of Gastroenterology and Endoscopy, St. John Hospital and Medical Center, Detroit, MI, USA; ^4^Section of Surgical Oncology, St. John Hospital and Medical Center, Detroit, MI, USA

## Abstract

Schwannomas are generally slow growing asymptomatic neoplasms that rarely occur in the GI tract. However, if found, the most common site is the stomach. Gastrointestinal stromal tumors (GISTs) are the most common mesenchymal tumors of the gastrointestinal tract, and 60–70% of them occur in the stomach. Owing to their typical presentation as submucosal neoplasms, gastric schwannomas and GISTs appear grossly similar. Accordingly, the differential diagnosis for a gastric submucosal mass should include gastric schwannomas. Furthermore, GI schwannomas are benign neoplasms with excellent prognosis after surgical resection, whereas 10–30% of GISTs have malignant behavior. Hence, it is important to distinguish gastric schwannomas from GISTs to make an accurate diagnosis to optimally guide treatment options. Nevertheless, owing to the paucity of gastric schwannomas, the index of suspicion for this diagnosis is low. We report a rare case of gastric schwannoma in 53-year-old woman who underwent laparoscopic partial gastrectomy under the suspicion of a GIST preoperatively but confirmed to have a gastric schwannoma postoperatively. This case underscores the importance of including gastric schwannomas in the differential diagnosis when preoperative imaging studies reveal a submucosal, exophytic gastric mass. For a gastric schwannoma, complete margin negative surgical resection is the curative treatment of choice.

## 1. Introduction

Mesenchymal tumors of the gastrointestinal (GI) tract are mainly comprised of a spectrum of spindle cell tumors which include gastrointestinal stromal tumors (GISTs), leiomyomas or leiomyosarcomas, and schwannomas [1]. Among these neoplasms, GISTs are the most common and a great majority (60–70%) of them occur in the stomach [2, 3]. Schwannomas, contrastingly, are generally slow growing asymptomatic neoplasms that rarely occur in the GI tract. However, if found, the most common site is the stomach, accounting for 0.2% of all gastric tumors [4, 5]. Accordingly, the main differential diagnosis of a gastric schwannoma is a GIST.

Owing to their typical presentation as submucosal neoplasms with spindle cell histology, gastric schwannomas and GISTs appear grossly similar [2, 6]. Both gastric schwannomas and GISTs occur predominantly in middle-aged persons [1, 5]. They also appear to have no distinct clinical features [1, 4, 7]. However, the prognosis for gastric schwannomas and GISTs is very different. As reported by Daimaru et al., in 1988, gastric schwannomas are benign tumors with an excellent prognosis [5, 6, 8], whereas 10–30% of GISTs have malignant behavior [1, 2, 9]. Hence, it is important to make an accurate diagnosis to optimally guide treatment options.

Nonetheless, a diagnostic difficulty exists in that preoperative conventional imaging techniques (i.e., sonography, endoscopy, and computed tomography), although helpful, cannot always provide enough information to differentiate between these two tumors. Indeed, radiologic and endoscopic findings are nonspecific [8, 10]. Furthermore, owing to the rarity of gastric schwannomas, there is limited data in the medical literature about the imaging appearance of this neoplasm. Ultimately, the definitive diagnoses of GISTs and gastric schwannomas require immunohistochemical studies which only can be performed on the surgical specimen.

In this paper, we present a 53-year-old woman with a gastric mass who underwent laparoscopic partial gastrectomy under the suspicion of a GIST preoperatively but confirmed to have a gastric schwannoma postoperatively.

## 2. Case Report

A 53-year-old woman was referred to surgical oncology for a gastric mass. The reason for her initial visit was chest pain. She only complained of intermittent gastric discomfort and dysphagia in response to solids. Her medical history was significant for hypertension, hyperlipidemia, diabetes mellitus, hypothyroidism, and uterine cancer, which was successfully treated with robotic hysterectomy 5 years prior.

**Figure 1:**
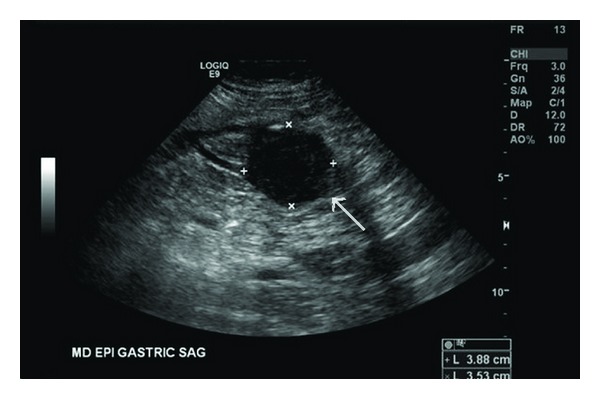
Transabdominal sonogram reveals a round and well-defined mass in the stomach.

**Figure 2:**
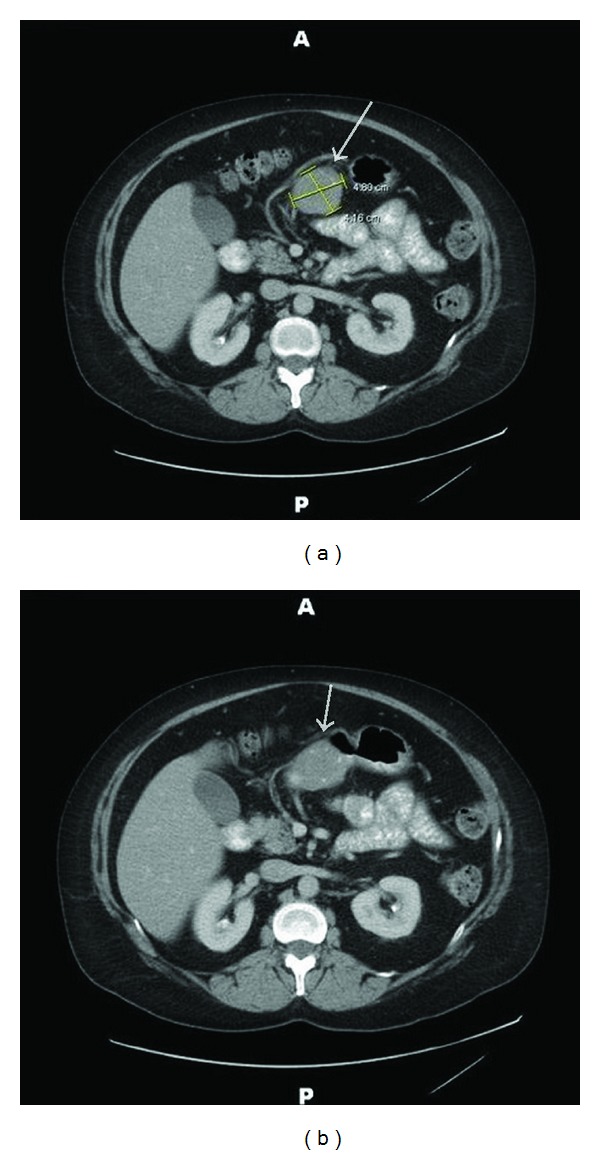
Contrast-enhanced CT showing a round, well-defined, and homogeneously enhancing mass arising from the antrum of the stomach.

During the chest pain assessment, a gastric mass was incidentally detected on abdominal ultrasound (Figure 1). A subsequent contrast-enhanced CT scan showed a homogenous exophytic mass, measuring 4.8 × 4.2 × 3.6 cm and arising from the antrum of the stomach (Figure 2). The overlying mucosa was smooth with a few minute calcifications. There was no evidence of any other abnormalities.

**Figure 3:**
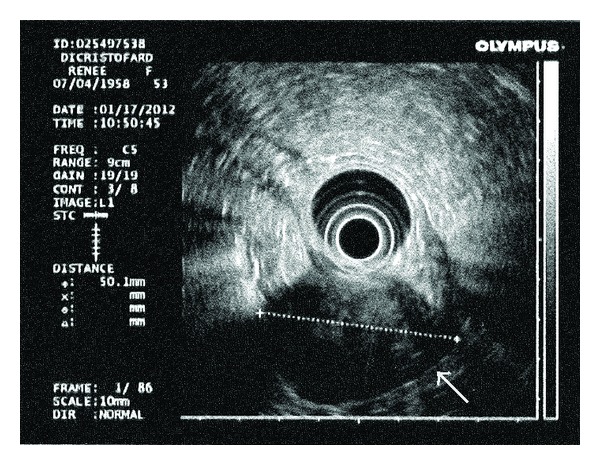
Endoscopic ultrasonogram: a 5 cm hypoechoic inhomogeneous mass lesion with calcification is seen. The mass appears to arise from the muscularis propria.

The patient then underwent an esophagogastroduodenoscopy (EGD), and a submucosal mass lesion was confirmed in the gastric antrum with normal overlying gastric mucosa. Biopsy specimens obtained at the endoscopy yielded only unspecific signs of mild inactive chronic inflammation without evidence of a malignancy. To facilitate the evaluation, we performed an endoscopic ultrasound (EUS) examination, which depicted a hypoechoic heterogeneous mass lesion with calcifications located in the gastric antrum (Figure 3). The mass had cystic spaces and appeared to arise from the muscularis propria, and there was no perigastric lymphadenopathy. EUS-guided fine needle aspiration (FNA) was then performed. The aspirate smears showed rare spindle cell tissue fragments with bland cytomorphological features. These findings appeared most consistent with a GIST.

**Figure 4:**
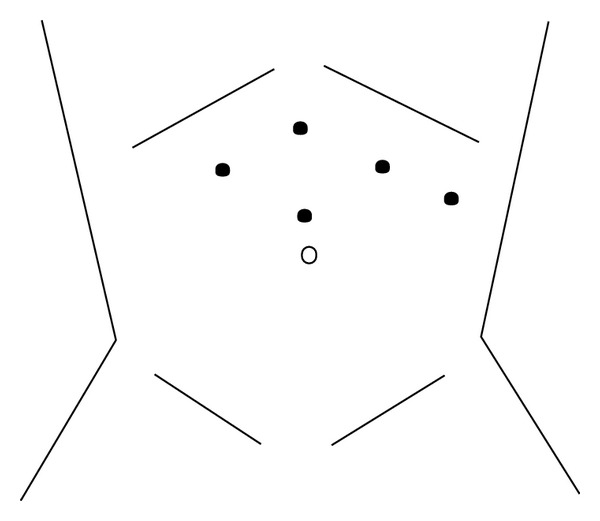
Schematic diagram showing trocar positions: three 5 mm trocars (right subcostal, subxiphoid, left lateral), one 10 mm trocar (left subcostal), and one 15 mm trocar (supraumbilical) are used.

**Figure 5:**
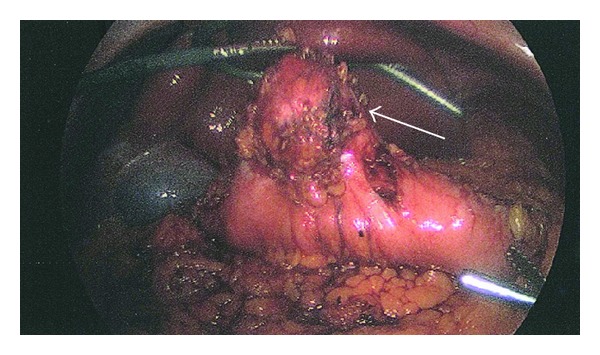
Macroscopic inspection during laparoscopic surgery shows a large exophytic mass along the greater curve in the antrum of the stomach.

After presenting the case at our Gastrointestinal Multidisciplinary Tumor Board Conference, a consensus was reached to proceed with resection. The patient was counseled about the surgical options and offered an elective laparoscopic partial gastrectomy. After informed consent was obtained, the patient was taken to the operating room where she was placed in the supine position under general endotracheal anesthesia. The abdomen was prepped and draped in sterile fashion. Pneumoperitoneum was achieved at 15 millimeters of mercury, and four additional trocars were placed under direct vision as shown in Figure 4. The stomach was mobilized by opening the gastrocolic ligament. Following mobilization of the greater curve, we clearly identified a large exophytic mass along the greater curve in the gastric antrum close to the pylorus. We isolated the mass from the stomach and actually suspended the mass from the stomach with laparoscopic Babcock atraumatic graspers (Figure 5). Due to the close proximity of the mass to the pylorus, we placed a 32-Fr boogie in the stomach and then used an endovascular gastrointestinal anastomosis (GIA) stapler across the base of the mass in viable stomach ensuring a negative margin. We then retrieved the specimen (the mass with a portion of the stomach) through an endocatch bag through the supraumbilical port and sent to pathology for analysis. The rest of the abdominal cavity was visualized, and there was no other evidence of abnormalities. The patient had a brief uneventful recovery.

**Figure 6:**
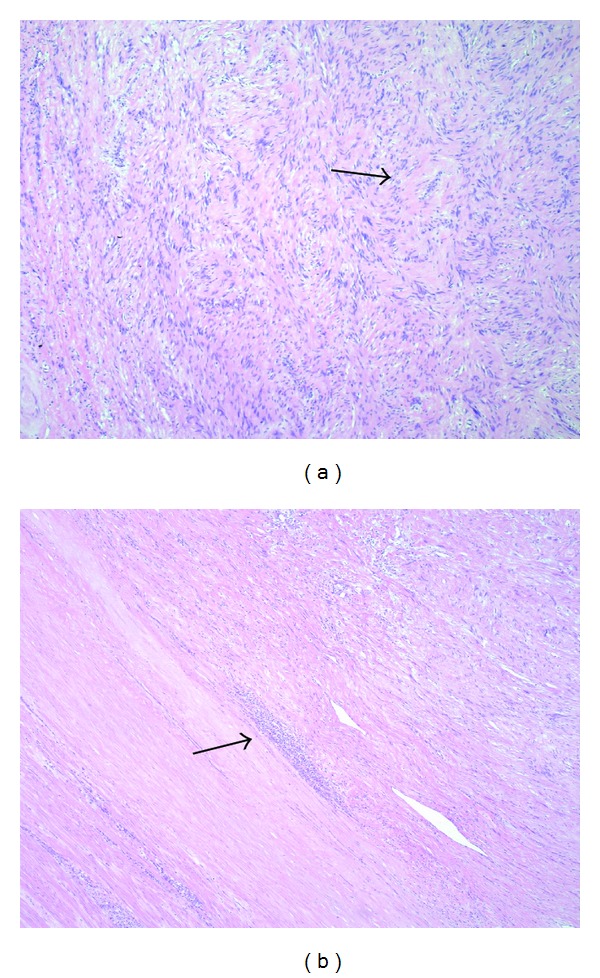
(a) Spindle cell proliferation with relatively bland cytology and focal nuclear palisading (*arrow*) (H&E, ×200); (b) lymphocytic cuffing (*arrow*) at the peripheral part of the tumor is a common feature (H&E, ×100).

**Figure 7:**
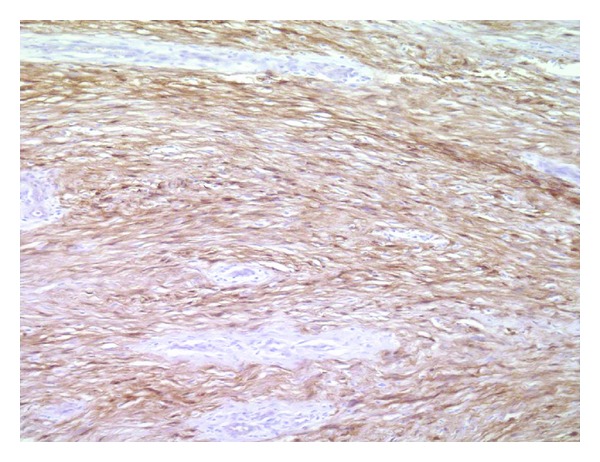
The tumor cells are positive for S-100 protein (immunostaining of S-100 protein, ×200).

**Figure 8:**
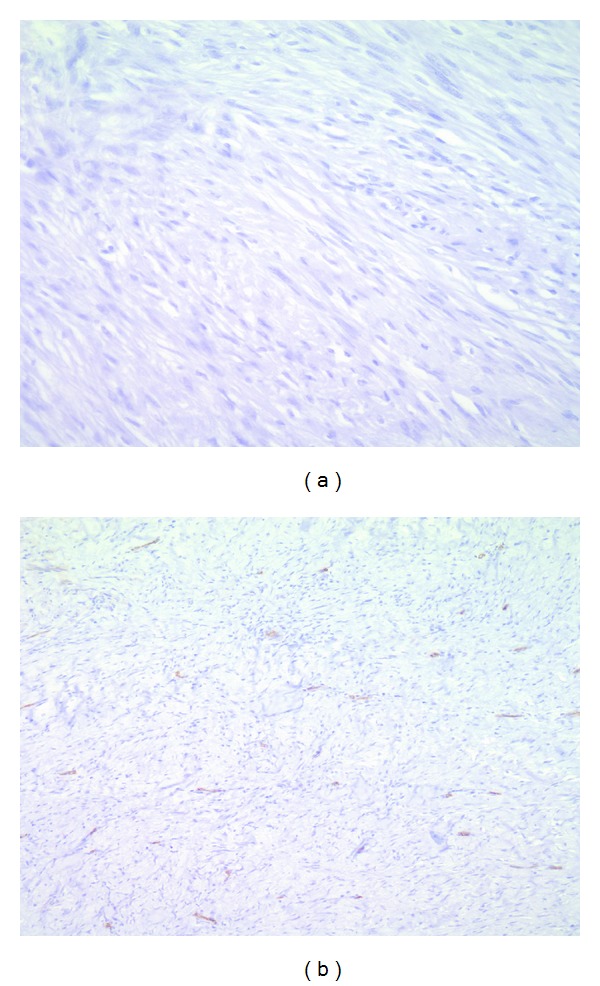
The tumor cells are negative for CD 117 immunostain ((a) ×200) and CD 34 immunostain ((b) ×200).

The final pathologic study revealed that the resected neoplastic mass was comprised of spindle cells of varying cellularity with relatively bland cytology and focal nuclear palisading (Figure 6(a)). There was lymphocytic cuffing at the periphery of the tumor (Figure 6(b)). The neoplastic cells were immunoreactive with S-100 protein (Figure 7), but lacked immunoreactivity with CD 117 (Figure 8(a)), CD 34 (Figure 8(b)), smooth-muscle actin and desmin. The histopathologic features and immunohistochemical staining pattern were consistent with a gastric schwannoma.

## 3. Discussion

Schwannomas, also known as neurilemmomas or neurinomas, are benign neurogenic tumors, originating from Schwann cells, which normally wrap around the axons of the peripheral nerves. Theoretically, schwannomas can develop anywhere along the peripheral course of nerve. However, they most commonly occur in the head and neck but rarely in the GI tract [11].

Gastric schwannomas, the most common GI schwannoma, account only for 0.2% of all gastric tumors, and principally involve the submucosa and muscularis propria [4–6]. They grow slowly and exophytically and are usually asymptomatic. Because of this indolent growth pattern, as with our case, these tumors often discovered incidentally via cross-sectional imaging or endoscopy [8, 11]. If symptomatic, the most common presenting symptom is upper GI bleeding, which may be secondary to the growing submucosal mass compromising the blood supply to the overlying mucosa. The mucosa overlying the mass may then ulcerate secondary to ischemia or from a reduced tolerance to the gastric acidity [4, 10, 11].

For a gastric submucosal mass, the main differential diagnosis is a GIST. Although rare, gastric schwannomas are also a primary GI mesenchymal tumor [6]. Most importantly, all published data to date indicate that GI schwannomas are benign neoplasms with excellent prognosis after surgical resection [5, 6, 8]. Therefore, the differential diagnosis for a gastric submucosal mass should include gastric schwannomas, for it is important to distinguish gastric schwannomas from GISTs. Nevertheless, owing to the paucity of cases, the index of suspicion for this diagnosis is low.

The differentiation between gastric schwannomas and GISTs can be difficult preoperatively. While preoperative imaging studies, such as sonography, endoscopy, and CT, can demonstrate the presence or extent of invasion, none of these modalities have shown any distinct features unique to these neoplasms [10–13]. Furthermore, due to the rarity of gastric schwannomas, there is limited data in the medical literature about the imaging features of this neoplasm.

In 2005, Levy et al. reported that gastric schwannomas are uniquely different from other schwannomas in that they show homogeneous attenuation on CT and that degenerative changes such as cystic changes are uncommon [12]. In addition, they suggested that the homogenous enhancement pattern may aid in differentiation of gastric schwannomas from GISTs, which frequently show heterogeneous enhancement due to degenerative changes. Later, in 2008, Hong et al. reported similar findings after studying 16 cases of gastric schwannomas at their institution: 13 cases showed a homogeneous enhancement pattern (81%), and cystic changes were seen in 2 cases (13%) [13]. Our case, which was immunohistochemically diagnosed as a gastric schwannoma postoperatively, had uncommon cystic changes and did not enhance intensely, although appeared as a homogeneous mass.

Consistent with the CT findings, endoscopic ultrasonogram of our case depicted a heterogeneously hypoechoic mass. It seemed that the cystic changes caused inhomogeneous echoes, and the dense composition of spindle cells caused the lower echogenicity [10]. Endoscopic tissue biopsies also yielded inconclusive results. As shown in this case, endoscopic biopsy may not be adequate for definite diagnosis because mucosal abnormalities are rarely observed in these submucosal tumors [8, 11]. 

Despite strong morphological similarities, GI mesenchymal tumors are heterogeneous in their immunophenotypes. In the past, gastric schwannomas were included in the GIST category [6]. In 1988, Daimaru et al. successfully identified schwannoma as a primary GI tumor based on the positive S-100 stain [5, 6]. GIST also became a distinct GI cancer diagnostic category when it was discovered that nearly all GIST cells express c-kit protein [1, 2]. Before the recognition of S-100 antigen and c-kit antigen in gastric schwannomas and in GISTs, respectively, these neoplasms were most often classified as leiomyoma, leiomyosarcoma, or gastrointestinal autonomic nerve tumor [1, 2, 5, 6]. With the advent of immunohistochemical staining techniques and ultrastructural evaluation, it is now possible to identify these neoplasms based on their distinct immunophenotypes. Gastric schwannomas are positive for S-100 protein and negative for c-kit; conversely, GISTs are positive for c-kit and negative for S-100 protein. Our case fulfilled the immunohistochemical diagnosis for a gastric schwannoma.

In summary, this case underscores the importance of including gastric schwannomas in the differential diagnosis when preoperative imaging studies reveal a submucosal, exophytic gastric mass. Thus far, the majority of previous series in the literature addressing gastric schwannomas regard these neoplasms as uniformly slow growing benign tumors. Owing to subclinical tumor growth, the diagnosis is usually delayed. However, to date, there is no clear evidence in the literature to suggest that gastric schwannomas have malignant potential. Additionally, recurrent disease has been only observed after incomplete resection [4–6]. Therefore, when diagnosed or suspected, complete margin negative surgical resection, as in this case, is the curative treatment of choice.
